# Clinical nurse competence and its effect on patient safety culture: a systematic review

**DOI:** 10.1186/s12912-023-01305-w

**Published:** 2023-05-19

**Authors:** Rasha Abu Zaitoun, Nizar B. Said, Lila de Tantillo

**Affiliations:** 1 Head of the Continuing Nursing Education, nursing department, An Najah National University Hospital, Nablus, Palestine; 2 Department of Nursing and Midwifery, Faculty of Medicine and Health Sciences, An Najah National University, Nablus, Palestine; 3 Keigwin School of Nursing, Jacksonville University, Brooks Rehabilitation College of Healthcare Sciences, 2800 University Boulevard North, Jacksonville, FL 32211 904.256.8955 USA

**Keywords:** Patient safety, Nursing competence, Safety culture

## Abstract

**Background:**

Unsafe health practices are one of the leading causes of disability and even death. Competent nurses are crucial to ensure safe and high-quality healthcare services. The patient safety culture is concerned with internalizing safety beliefs, values, and attitudes, translating them into healthcare practices, and committing to maintaining an error-free health environment. A high level of competence ensures the achievement and compliance with the safety culture goal. This systematic review aims to identify the relationship between the level of nursing competence and the safety culture score and perception among nurses at their workplace.

**Methods:**

Four international online databases were searched to find relevant studies published between 2018 and 2022. Peer-reviewed articles using quantitative methods, targeting nursing staff, and written in English were included. After reviewing 117 identified studies, 16 full-text studies were included. The PRISMA 2020 checklist for systematic reviews was used.

**Results:**

Evaluation of the studies indicates safety culture, competency, and perception were assessed using various instruments. Safety culture was generally perceived as positive. No unique and standard tool has been developed to investigate the effect of safety competency on the perception of the safety culture in a standardized way.

**Conclusions:**

Existing research provides evidence of a positive correlation between nursing competence and patient safety score. Future research is recommended to investigate ways to measure the effect of nursing competency level on safety culture in healthcare institutions.

**Supplementary Information:**

The online version contains supplementary material available at 10.1186/s12912-023-01305-w.

## Background

Unsafe health practices are highly regarded to cause disability and death. It is estimated that the chance that unsafe practice can cause harm for the patient is 1 in 300 chance [1]. Nearly 400,000 deaths occur annually in the United States due to several reversible adverse events, such as medication error, infection transmission, and fall events [2]. In addition, poor quality care can cause death and a global health burden [3]. Patient safety is a health care discipline that evolved as a result of the increasing sophistication of health care systems and the increasing in adverse outcomes in health-care facilities. Its goal is to avoid and decrease risks, mistakes, and harm to patients while providing health care. Therefore, reliable, safe, equitable, effective, and highly standardized patient-centered care has become the ultimate goal of all health care institutions worldwide [4]. Besides that, patient safety culture focuses on organizational culture issues related to patient safety, patient safety culture is concerned with internalizing safety beliefs, values, and attitudes, translating them into health care practices and commitment to maintaining an error-free health setting and emphasizing reporting culture [5].

Competent nurses are key contributors to maintaining safe and effective health care services by integrating knowledge, skills, and attitudes that enable them to adapt to dynamic health environments [6]. Nurses are often the primary point of contact for patients and are responsible for ensuring that their needs are met. By providing patient-centered care, nurses can help create a culture that prioritizes patient safety. In addition, they can act as advocates for patient safety, promoting a culture of safety within the organization and encouraging others to do the same. Nurses should be given the power to make decisions about patient care and safety, as well as to report any harmful conditions or concerns. They can indeed act as role models for other healthcare professionals, emphasizing the importance of patient safety and providing a good example for others to follow.

Patient safety competencies are a core competency in the continuum of professional development activities that protect patients from unnecessary risks and hazards [7]. A high level of competence promotes the achievement and compliance with the patient safety goal.

Several studies found that patient safety culture and nurse safety competency are affected by many factors. For example, workplace regulations and climate, nursing fatigue, satisfaction, stress, demographics, type of health institution type, teamwork and learning opportunity, specialty, degree of bedside involvement, and job description are all factors that affect safety culture [5, 8–11].

Any improvement strategies to modulate these factors are unnegotiable. However, a better understanding of nurse competence and patient safety culture is essential to improve safe practice and professional development and minimize adverse events [12]. Therefore, this systematic review aimed to evaluate the literature concerning the relationship between self-reported competencies and the perception of patient safety among nurses in their workplace. In addition, to understand how related studies evaluated nurses’ core competencies and safety culture.

## Methods

### Study design

The present systematic review was designed and conducted from April 1, 2022, and April 11, 2022, by the electronic search from January 1, 2018, through May 1, 2022. This date period was chosen after agreement between the author to find the (five years) recent evidence regarding the respected review topic. The current systematic review was framed by SPIDER [13] with nurses as the Sample (S); clinical competence as the Phenomenon of Interest (PI); Design (D) as quantitative or mixed-methods studies; Evaluation (E) as covering assessments of patient safety culture; and Research Type (R) as referring to all types of studies with the exception of case studies and review articles. The protocol was performed based on the guidelines of the Preferred Reporting Items for Systematic Review and Meta-Analyses Protocol (PRISMA-P) [14].

### Search strategy and data sources

Four online databases including MEDLINE (via PubMed), CINHAL (via EBSCOhost), Scopus (via Elsevier), and Embase were searched for published studies that describing the relationship between nurse competencies and patient safety culture. Search terms were developed based on experience and keywords from similar research. The search was structured using Boolean operators (AND, OR) and consisted of MeSH terms and free terms related to nursing, patient safety, competency, and safety culture. The term ‘OR’ was used between keywords or comparable MeSH phrases; meanwhile, the Boolean operator ‘AND’ was used to connect phrases or keywords with different meanings to refine the search (see Appendix A). Relevant studies were identified by two reviewers (R.A. and L.T.) independently, and search algorithm varied according to the specifications of each database. To identify the additional relevant studies being lost in the database search, we checked the references of the selected publications.

### Inclusion and exclusion criteria

In this review, studies were included if they are using the quantitative approach mainly; targeting nursing staff; published in English language, and full text available. The exclusion criteria were studies in the form of letters, editorials, essays, case studies, comments or narrative, systematic reviews, and conference abstracts; studies focus on nursing students only; and studies of pre-hospital and ambulatory care.

### Study selection and quality assessment

After preliminary selection of studies by the first reviewer (R.A) and their verification by the last reviewer (L.T.) the duplicated studies were excluded. Two reviewers (R.A. and L.T.) independently screened the titles, abstracts and full text of the studies.

The Mixed Methods Appraisal Tool (MMAT) [15] was used to evaluate the quality of the studies included in this review. With the MMAT, two basic screening questions must be asked first to determine whether or not the quality appraisal for a specific study will be continued. The first question is whether the related qualitative, quantitative, or mixed-methods study has clear research questions or objectives. The second question is whether the method of data collection addresses the research questions or objectives. When these initial screening questions are answered positively, qualitative (QUAL) or quantitative (QUAN) studies can be rated as follows: ‘*’ (25%) for one met criterion, ‘**’ (50%) for two, ‘***’ (75%) for three, or ‘****’ (100%) for the highest quality study. Studies with a rating of ** or higher were considered to be of acceptable quality for this review and were included for further analysis.

### Data extraction

Data extraction table included: author(s) and publication year, country, design of study, method of data collection, quality assessment. Measurement Tools for Safety Culture and Nursing Competency and main findings focusing on Patient Safety Competency and the Patient Safety Culture. In this stage two authors (R.Z. and L.T.) independently extracted data from the included studies. In case of disagreement between two reviewers (R.Z. and L.T.), a third reviewer (N.S) was involved to make a final decision.

### Data synthesis

An iterative narrative synthesis technique was used for data analysis [16] to identify key themes from every study that would adequately and accurately reflect the findings about clinical nurse competence and patient safety culture. The narrative synthesis was divided into four stages: (1) identifying a frame of how findings from related studies work, why and for whom; (2) synthesizing themes through an iterative process of comparing and examining findings from the included studies; (3) trying to explore themes’ relationships; and (4) evaluating the synthesis’s robustness.

## Results

### Results of the search strategy

**Fig. 1:**
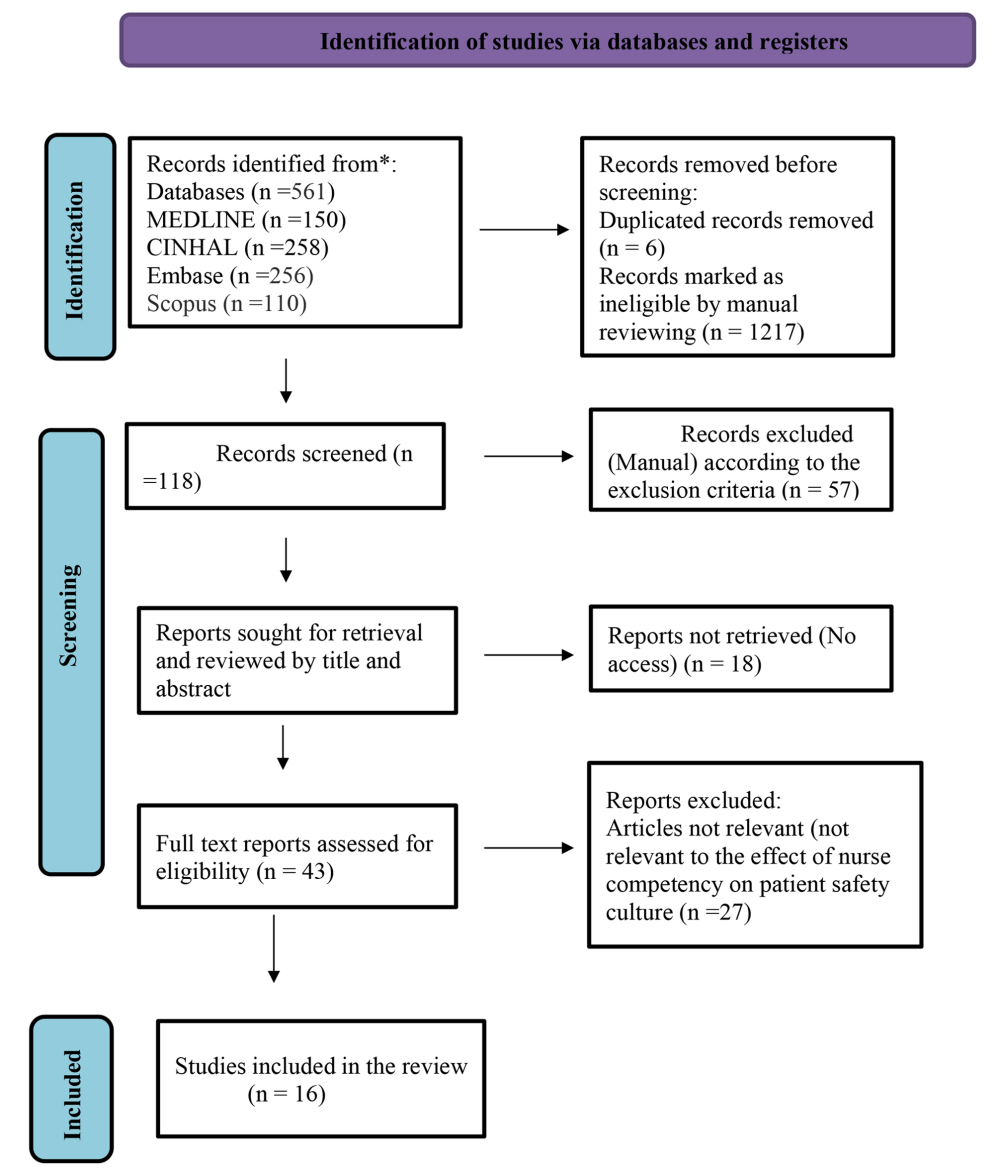
PRISMA flow diagram of the systematic review

Through the flow of PRISMA-P, the search process found 1341 potentially relevant studies; initial screening revealed that 6 records were duplicated. After excluding the irrelevant studies by title and abstract review (1217) and applying exclusion criteria (57), reports not retrieved related to access issues (n = 18). Full text screening showed that 27 were not relevant. Finally, 16 studies remained and were included in the analysis (Fig. 1).

### The design of the reviewed studies

Of the 16 qualifying studies, 14 were cross-sectional descriptive studies, one applied a quasi-experimental, pretest-posttest design [17], and one study used a mixed method approach [18]. Three out of the sixteen studies were conducted in South Korea [19–21], Canada [19] Three studies originated from Saudi Arabia [9, 22, 23]. Two studies from Iran [24, 25]. In addition, a study was conducted in each Australia [26], Belgium and Palestine [27], Brazil [28], Jordan [29], Spain [18], England [17], and China [11]. The aforementioned research studies were published from 2019 to 2022 in different journals. Summary of the characteristics of included studies are depicted in Table 1.

**Table 1:** Summary characteristic of included studies

Author (year)	Country	Design	Participants (n)	Method of data collection	Quality assessment
**(Alshehry, 2022)**	Saudi Arabia	A descriptive, cross-sectional design	Nurses (320)	Survey questionnaire administration	****
**Connell et al.** **(2021)**	Australia	A cross-sectional design	Doctors (44) Nurses (119)	Survey administration	****
**(Halabi et al., 2021)**	Saudi Arabia	A cross-sectional design	Nurses (469)	Survey administration	****
**(Habibi Soola et al., 2022)**	Iran	Cross-sectional, correlational design	Emergency nurses (254)	Questionnaire administration	****
**(Han & Roh, 2020)**	Korea	A descriptive, correlational, cross-sectional design	Emergency nurses (200)	Survey questionnaire administration	***
**(Han et al., 2020)**	Korea	Cross sectional	Nurses (212)	Survey administration	****
**(Huh & Shin, 2021)**	Canada	A descriptive, cross-sectional design	Nurses in geriatric hospitals (186)	Survey administration	****
**(Khamaiseh et al., 2020)**	Jordan	A cross-sectional, descriptive study	Primary health-care nurses (644)	Survey administration	***
**(Kakemam et al., 2021)**	Iran	A nationwide cross-sectional study	Nurses (2295)	Survey administration	***
**(Letourneau & McCurry, 2019)**	England	A quasi-experimental with Pretest-posttest design	Nurses (64)	Pretest-posttest survey administration	****
**(Lousada et al., 2020)**	Brazil	A cross-sectional study	Multiprofessional Home Care Team (147)	Survey questionnaire administration	****
**Mahsoon and Dolansky (2021)**	Saudi Arabia	Correlational, cross-sectional design	Registered nurses (84)	Survey administration	***
**(Najjar et al., 2018)**	Belgium and Palestine	Observational, cross-sectional study	Nurses, head nurses, nursing aid staff, physicians, pharmacists, and other healthcare staff (2836)	Survey administration	***
**(Peñataro-Pintado et al., 2022)**	Spain	Mixed method	- Delphi method: clinical safety experts (13) and perioperative nurses (25) - Survey administration: nurses (415), experts in clinical safety (9), Postgraduate studies in perioperative nursing (56)	- Delphi method: focus group - Survey administration	***
**(Son et al., 2019)**	South Korean	A cross-sectional descriptive design	Nurses (364)	Survey administration	****
**(Yan et al., 2021)**	China	Cross sectional	Registered nurses (451)	Survey questionnaire administration	***

### The sampling technique

Given the sampling technique, eight articles used the convenience sampling technique [9, 11, 19, 22, 23, 25, 26, 30]. The highest sample size was 2,836 participants in the study by Najjar et al. [27], while the least was 56 participants in [28] study.

### Methodological quality of included studies

The assessment of the methodological quality of the included studies is presented in Table 2. After quality assessment, nine studies graded as ‘****’ (100%) and seven studies as ‘***’ (75%) quality using MMAT. Research conducted or published during the COVID-19 outbreak period [9, 11, 18–26, 28, 29] received additional review to ensure it met rationale for inclusion and standards for quality, to ensure rigor in response to the substandard work proliferating during this period [31].

**Table 2:** Measurement tools and main findings of included studies

Author (year)	Measurement Tools	Main findings
**Alshehry** **(2022)**	Health Professional Education in Patient Safety Survey (H-PEPSS) - Healthcare Conflict Scale (HCS)	“Mistrust of motivations” showed the most conflict, while “contradictory communication” was regarded as the least. There was a considerable disparity between the perceived conflict and the various hospital units where nurses worked. “Communicating effectively” had the greatest patient safety competence, while “working in teams with other health professionals” had the lowest safety competence. Nurses who experienced “mistrust of motivations” and “contradictory communication” had lower self-reported patient safety competence.
**Connell et al.** **(2021)**	- Safety Attitudes Questionnaire (SAQ) - Safety Climate Survey (SCS)	Nurses rated the organization’s commitment to patient safety higher than doctors in all remaining attitudinal domains. Nurses and doctors acknowledge that fatigue, increased workload and stress recognition negatively impacts upon patient safety.
**(Halabi et al., 2021)**	Nurse Professional Competence (NPC) scale short version	There were significant relationships between self-reported professional competence and the quality of nursing care, patient safety, nurse’s characteristics, and experience. The study assessed the RNs’ professional competence related to their work in different clinical areas. The highest scored area of competence was Nursing Care, and Value-based while the lowest scored areas were Care Pedagogics, Development, Leadership and Organization of Nursing Care.
**(Han & Roh, 2020)**	- Brief- TeamSTEPPSTM Teamwork Perceptions Questionnaire - Psychological Safety Subscale of the Team Survey Scale	The patient safety competency affected significantly by situation monitoring, reporting of patient safety adverse events, frequen-cy of night shifts, and psychological safety.
**(Han et al., 2020)**	- Patient Safety Competency Self-Evaluation Tool - Health Professional Education in Patient Safety Survey (H-PEPSS)	Patient safety dimension of openness in communication scored high in the study and was significantly correlated with decreased rates pressure sore and falling down also the dimension of team work in patient safety competency were significantly was significantly associated with decrease in in ventilator-associated pneumonia.
**(Kakemam et al., 2021)**	- Hospital Survey on Patient Safety Culture (HSOPSC) - Adverse event evaluation report	Nurses’ perception regarding patient safety culture was low and the perceived occurrence of adverse events was high. Higher level of nurses’ perception of patient safety culture was associated with lowered occurrence of adverse events.
**(Khamaiseh et al., 2020)**	- Safety Attitudes Questionnaire (SAQ – Short Form)	The six safety culture domains had an average positive response percentage that ranged from 58.54–75.63%. Job satisfaction had the greatest average positive response rate, while management perspectives had the lowest.
**(Letourneau & McCurry, 2019)**	The Nursing Quality and Safety Self- Inventory (NQSSI)	The Nursing Quality and Safety Self-Inventory was a valid and reliable instrument for measuring changes in quality and safety competence in newly licensed registered nurses NLRN and the transition to practice programs TPPs were effective to advance trust in quality and safety competencies
**(Lousada et al., 2020)**	- Safety Attitudes Questionnaire (SAQ)	Among the participants, being male and had clinical experience of three to four years highly scored on Safety Climate, Job Satisfaction, Teamwork Climate. The dimension of Perception of management and Working conditions had the lowest scores.
**Mahsoon and Dolansky (2021)**	- Nurses’ Attitudes and Skills Safety scale (NASUS) - Patient safety attitudes, skills, and knowledge (PS-ASK) scale - The hospital survey on patient safety culture (HSOPSC) - Safety performance (self-reports) - Systems thinking scale (STS)	The safety competency subscales of skill knowledge and attitude showed acceptable internal consistency and reliability based on Cronbach’s alpha coefficients of 0.80 or greater. Safety competence was predicted by systems thinking. The baccalaureate educational level, completion of safety training and the safety culture item of ‘Mistakes have led to positive changes here’ were predictive of the skill subscale.
**(Najjar et al., 2018)**	Hospital Survey on Patient Safety Culture (HSOPSC)	The results showed that HSOPSC was a valid tool to assess patient safety culture. The dimension of non-punitive response to error had no association with any of the outcome measures in Belgium. The overall perception of safety was highly predicted by hospital management support in Palestine and by staffing in Belgium while the number of events was largely predicted by feedback and communication in both countries.
**(Peñataro-Pintado et al., 2022)**	Questionnaire of Perioperative Nursing Safety Competencies (Spanish acronym is CUCEQS© questionnaire)	The study showed that Perioperative Nursing Safety Competencies instruments CUCEQS© was a valid tool for measuring the perceived level of competency by the perioperative nurses in surgical patient safety.
**(Son et al., 2019)**	- Health Professional Education in PS Survey - Adverse Nurse Outcomes	In this study, the patient safety competencies had inverse relationship with the increase in working hours and were significantly associated with adverse nurse events specially having more than 40 working hours.
**(Yan et al., 2021)**	Patient Safety Competency Nurse Evaluation Scale (PSCNES)	The overall patient safety culture score among associated degrees nurses in the study was moderate. The highest scores were achieved for the dimensions of clinical practice and safety risk management and the domains of patient safety culture and patient-centered care scored low. Nurses who attended the patient safety based training scored higher in all patient safety culture than nurses who did not attend.

### Measurement tools for Safety Culture and nursing competency

The construct concepts of patient safety culture, safety climate, and patient safety competency were operationally measured using various tools or instruments throughout the sixteen studies. Nursing safety competency was measured using the Patient Safety Competency Self-Evaluation Tool, the Health Professional Education in Patient Safety Survey (H-PEPSS) [21], and the Nurses’ Attitudes and Skills Safety scale, the latest version of NASUS [23]. Furthermore, AS Alshehry [22] used the Health Professional Education in Patient Safety Survey (H-PEPSS) to assess safety competency. Halabi et al. [9] used the short version of Nurse Professional Competence (NPC).The self-reported Patient Safety Competency Nurse Evaluation Scale (PSCNES) was used by Yan et al. [11].

Three studies applied the Hospital Survey on Patient Safety Culture (HSOPSC) developed by the Agency for Healthcare Research and Quality to measure safety culture [21, 27, 32]. The safety climate was measured using the Safety Attitudes Questionnaire (SAQ) and the Safety Climate Survey (SCS); [26, 28, 29] (Table 2).

### The patient safety culture

Patient safety culture was mentioned in the title of six studies [21, 23, 27, 28, 32, 33]. There was considerable variation of how this concept was perceived among nurses for example, LM Lousada, FC da Silva Dutra, BV da Silva, NLL de Oliveira, IB Bastos, PF de Vasconcelos and R de Carvalho [28] in his study found that professionals working in home care services perceived higher scores related to safety culture compared with those working in primary care services. The accredited primary center in Jordan had an average positive response rate in some safety cultures ranging from 58.54 to 75.63% [33], A total of 32 Iranian teaching hospitals out of 150 reported poor patient safety culture [32]. On the other hand, and with regard to the safety climate, the study by CJ Connell, S Cooper and R Endacott [26] revealed that novice-competent nurses in Australian emergency departments rated the safety climate higher than expert nurses in all domains except stress recognition.

### Patient safety competency

Six studies examined patient safety competency and several studies connected safety competencies with the domains of safety culture. For example, L Yan, L Yao, Y Li and H Chen [11] assessed the safety competence scores of Chinese nurses with associate degree and they scored moderate level. A Habibi Soola, M Ajri-Khameslou, A Mirzaei and Z Bahari [25] found a positive correlation between safety competence and the dimension of team work, psychological safety, leadership, communication, mutual support, situation monitoring, and team structure. JH Han and YS Roh [20] found that the night shift among emergency nurses in Korean hospitals negatively affected safety competence and was significantly and positively correlated with the number of years of experience in the emergency department and number of reported adverse events by others.

In a medical city in Saudi Arabia, AS Alshehry [22] studied the correlation between the conflict between nurses and the patient, and safety competencies. The study revealed that nurses were highly competent in effective communication, but they had the lowest competency in ‘working in teams with other health professionals.’ Nurses perceived ‘mistrust of motivations’ and “contradictory communication” got the poorer self-reported safety competency.

## Discussion

The purpose of this study was to provide a systematic review of the literature investigating the relationship between nursing competencies and perception of patient safety among nurses in their workplace. In this systematic review, and after a thorough analysis of the entire manuscript of the retrieved articles, we selected and discussed sixteen articles based on their conformity with the inclusion criteria.

Nurse’ competence refers to the knowledge, skills, and abilities that nurses possess to provide safe and effective care to patients in many fields such as clinical, safety, communication, and leadership. This can include things like knowledge of clinical guidelines, critical thinking skills, and the ability to identify and respond to changes in a patient’s condition, how to manage conflict and communicate with patients and other health care team. A positive patient safety culture is one in which all members of the healthcare team are committed to providing safe care and are empowered to identify and report potential hazards. Research has shown that, specifically, safety nurse competence is positively associated with a positive patient safety culture [34]. Nurses who are competent in their practice are more likely to be engaged in the safety culture of their organization and more likely to identify and report safety concerns [11]. Additionally, nurses who possess the knowledge, skills and abilities required to provide safe care are less likely to make errors which can lead to adverse patient outcomes [35]. Overall, clinical nurse competence is a critical component of a positive patient safety culture, as it helps ensure that nurses are able to provide safe and effective care to patients and promotes engagement and active participation in the safety culture of the organization.

None of the reviewed articles explicitly explored the relationship between clinical or professional nursing competencies and the safety culture dimensions and how they affect each other. Furthermore, studies identified no specific comprehensive tool with high reliability and validity and mostly recommend assessing the relationship between nursing safety competency and the dimensions of safety culture. Rather, the included articles examined patient safety culture, health care safety climate, and other nursing competencies that influenced or affect safety climate independently. Most of the retrieved studies investigated the very specific competencies for nurses which were safety competency and the level of this competency varied among different clinical setting or nationality and range between poor and moderate level and this similar to SM Cho and J Choi [36] in their study investigated relationship between the three parts of safety competency and domains of patient safety culture among 343 registered nurses in an educational hospital in, South Korea and found that the safety competency was highly correlated with teamwork within units. Teamwork within and across units, supervisor or manager expectations, and each of the three patient safety competencies were strongly associated to attitudes, while teamwork within units and learning were significantly connected to skills. Knowledge was only significantly correlated with organizational learning.

The concentration on assessing and improving Safety competency for nurses instead of more general clinical nurse competence may due to that many health care institutions tend to adopt the principles of patients safety as their institutional goal and so try to improve nursing skills, knowledge and attitude related to patient safety and it would be better to assess the level of nurses’ culture of safety and their safety competency level in order to improve area of weakness or poor practices and reduce adverse events [37, 38].

### Measurement of patient safety culture

Measuring patient safety culture involves assessing the attitudes, perceptions, and behaviors of healthcare staff related to patient safety in a healthcare organization. There are several tools and survey instruments available to measure patient safety culture. In this review, two self-reported questionnaires were found to have been applied to understand the dimensions and scores of patient safety culture in health institutions. The Safety Attitude Questionnaire (SAQ) [28, 33] and the Hospital Survey on Patient Safety Culture (HSOPSC) [21, 23, 27, 32] were two assessment tools that employed Likert scales. However, in their study, G Alsalem, P Bowie and J Morrison [39] revealed that five instruments are used to assess the patient safety culture and climate in health institutions. Furthermore, these tools vary in their psychometric properties and scope.

The aforementioned survey instruments assess various aspects of patient safety culture, such as communication, teamwork, error reporting, and leadership. The results of these surveys can be used to identify areas of strength and areas for improvement in the patient safety culture of an organization.

In general, patient safety culture measurement is a continuous activity that should be conducted on a regular basis to assess success and identify areas for improvement. The information gathered through these surveys and other ways may be utilized to design and execute plans to improve the patient safety culture.

The most common study design among the studies was a questionnaire-based descriptive quantitative approach. Using different study designs, such as qualitative research and a variety of data collection methods, could help improve understanding of the safety culture and health care provider perceptions and would be required to address the existing relationship between safety culture and nursing competency [40].

### Measurement of safety nursing competency

Eight of the studies, or 50% of the literature, in this review showed that nurses were more competent in communicating effectively than working in teams with colleagues, and their overall safety culture score was positive. Likewise, LM Zabin, RSA Zaitoun and AA Abdullah [41] found that both organizational learning and continuous improvement, as well as cooperation within units, received the highest composite frequency of patient safety perception.

In the current findings, the reviewed literature mentioned four self-reported measurement tools for safety competency. Despite that, there was no consensus on the best tool for measuring safety competencies, and the lack of a key self-reported tool for measuring safety culture and linking it to nursing safety competency limited the ability to directly assess the effect of nursing competency on safety culture [42]. As a result, more research is needed to enrich the literature, improve the understanding of the effect of safety competency on safety culture scores among nurses, and help in providing more appropriate operational definition of safety culture and nursing safety competency [43].

Although we used broad keywords to search different online databases, the retrieved articles did not specifically discuss the relationship between the dimensions of the safety culture, perception, and competency in nursing safety. Additionally, the lack of a standardized tool to measure the concepts of safety culture and nursing safety competency made it difficult to find a robust number of targeted studies and limited our ability to find specific operational definitions to the concepts of safety culture and nursing safety competency that was used consistently across the literature.

Moreover, using self-reported surveys and relying on convenient sampling eased data collection and provide more objective data for many of the studies in this review. However, there were drawbacks that limited the generalizability and might not cover all aspects of the studies’ content. Therefore, future use of mixed designs with the use of qualitative methods is highly recommended to deepen the study issue and explore the unique relationship between the dimensions of the safety culture and nursing competency that can play a pivotal role in improving safety practices.

This review study recognizes the importance of conducting additional searches and reviews and broadening the scope of keywords used to search online databases to focus on the core of this study. In addition, it is recommended to investigate the effect of nursing competency on perceptions of dimensions of safety culture and to make valid comparisons between demographics and cultures.

More research would provide a better understanding and may have a greater clinical impact and aid in improving and delivering highly effective, safe, and efficient care. Furthermore, the findings would support synchronicity between academic clinical programs and nursing staff safety practices. For example, nursing students receive competency-based training that allows them to live in a safe environment and directly implement the dimension of safety culture [44].

### Limitations

This systematic review study has several limitations. First, we restricted the databases to four primary resources considered suitable for gathering eligible articles for the study purpose. The second is that the delimiters of this review included only articles in English, so some related articles may not have been included. Another limitation that should be highlighted is that we reviewed articles published between 2018 and 2022 to include the most recent data, but this also restricted the number of retrieved studies.

Another potential limitation was that the results of the reviewed studies could not be generalized. The articles were only published in peer-reviewed journals to ensure the high quality of evidence and the reported findings, which omitted many worthwhile studies such as grey and unpublished studies. Additionally, the study designs of the majority of the retrieved papers were descriptive, which restricts the generalizability of their findings and prevents access to many relevant studies. Therefore, it is recommended to conduct further systematic or integrative reviews that might include qualitative and descriptive studies as well as expand the inclusion criteria for other types of literature.

## Conclusions

This systematic review draws several conclusions from the sixteen reviewed articles. First, the study showed no specific tool to measure the safety culture and nursing safety competency dimensions. Additionally, no study explicitly discussed the effect of nursing safety competency on safety culture scores among nursing staff. However, most of the studies employed a questionnaire-based descriptive approach. Conducting more research with different study designs such as the experimental, qualitative, and longitudinal approaches may enhance the understanding and assist in constructing a valid and more reliable tool to measure the effect of nursing safety competency on safety culture. Second, rigorous research needed to establish a well-designed competency-based training program to improve safety scores among more diverse demographics and cultures is needed. The findings can motivate administrators to promote safety culture in different health care facilities, as well as increase professional awareness of the factors that impact safety culture and, consequently, patient safety. Finally, the key to improving safety competency for nurses is to create a culture of continuous learning and improvement, where nurses are encouraged and supported to develop their knowledge, skills and abilities in order to provide safe, high-quality care to patients.

## Electronic supplementary material

Below is the link to the electronic supplementary material.

Supplementary Material 1

## Data Availability

All data generated or analyzed during this study are included in this published article. The datasets used and/or analyzed during the current study available from the corresponding author on reasonable request.
