# Supplementary material for: Clinical nurse competence and its effect on patient safety culture: a systematic review
Source: BMC Nurs. 2023 May 19;22:173. doi: 10.1186/s12912-023-01305-w (PMC10196295; doi:10.1186/s12912-023-01305-w)
Supplement: Supplementary file 1 — Supplementary Material 1 [file 12912_2023_1305_MOESM1_ESM.pdf]

Appendix A

PubMed Search Strategy

((((((nurse*) AND (competency)) OR (competencies)) OR (competence)) AND (patient)) AND (safety culture)) OR (safety climate) Filters: Full text, Observational Study, English, from 2018 – 2022.

(((("nurse*"[All Fields] AND ("compete"[All Fields] OR "competed"[All Fields] OR "competences"[All Fields] OR "competencies"[All Fields] OR "competently"[All Fields] OR "competent"[All Fields] OR "competes"[All Fields] OR "competing"[All Fields] OR "mental competency"[MeSH Terms] OR ("mental"[All Fields] AND "competency"[All Fields]) OR "mental competency"[All Fields] OR "competence"[All Fields] OR "competency"[All Fields] OR "competent"[All Fields])) OR ("compete"[All Fields] OR "competed"[All Fields] OR "competences"[All Fields] OR "competencies"[All Fields] OR "competently"[All Fields] OR "competents"[All Fields] OR "competes"[All Fields] OR "competing"[All Fields] OR "mental competency"[MeSH Terms] OR ("mental"[All Fields] AND "competency"[All Fields]) OR "mental competency"[All Fields] OR "competence"[All Fields] OR "competency"[All Fields] OR "competent"[All Fields]) OR ("compete"[All Fields] OR "competed"[All Fields] OR "competences"[All Fields] OR "competencies"[All Fields] OR "competently"[All Fields] OR "competents"[All Fields] OR "competes"[All Fields] OR "competing"[All Fields] OR "mental competency"[MeSH Terms] OR ("mental"[All Fields] AND "competency"[All Fields]) OR "mental competency"[All Fields] OR "competence"[All Fields] OR "competency"[All Fields] OR "competent"[All Fields])) AND ("patient s"[All Fields] OR "patients"[MeSH Terms] OR "patients"[All Fields] OR "patient"[All Fields] OR "patients s"[All Fields]) AND ("safety

management"[MeSH Terms] OR ("safety"[All Fields] AND "management"[All Fields]) OR "safety management"[All Fields] OR ("safety"[All Fields] AND "culture"[All Fields]) OR "safety culture"[All Fields])) OR (("safety"[MeSH Terms] OR "safety"[All Fields] OR "safeties"[All Fields]) AND ("climate"[MeSH Terms] OR "climate"[All Fields] OR "climates"[All Fields] OR "climate s"[All Fields] OR "climatic"[All Fields] OR "climatically"[All Fields]))) AND ((fft[Filter]) AND (2018:2022[pdat]) AND (english[Filter]))
